# Short- and Long-Term Outcomes After Transcatheter or Surgical Aortic Valve Replacement in Patients With Chronic Lung Disease: An Analysis From the German Aortic Valve Registry

**DOI:** 10.1093/icvts/ivaf189

**Published:** 2025-08-18

**Authors:** Andreas Böning, Umniye Balaban, Eva Herrmann, Andreas Beckmann, Raffi Bekeredjian, Sabine Bleiziffer, Stephan Ensminger, Christian Frerker, Helge Möllmann, Thomas Walther, Timm Bauer

**Affiliations:** Department of Cardiovascular Surgery, University Hospital Giessen, 35392 Giessen, Germany; Goethe University Frankfurt, Institute of Biostatistics and Mathematical Modelling, Frankfurt, Germany; German Center for Cardiovascular Research, DZHK (Deutsches Zentrum für Herz-Kreislaufforschung), Partner Site Rhine-Main, Frankfurt am Main, Germany; Goethe University Frankfurt, Institute of Biostatistics and Mathematical Modelling, Frankfurt, Germany; German Center for Cardiovascular Research, DZHK (Deutsches Zentrum für Herz-Kreislaufforschung), Partner Site Rhine-Main, Frankfurt am Main, Germany; Department for Cardiac Surgery, Heart Center Duisburg, Duisburg, Germany; Department of Cardiology, Robert-Bosch Hospital Stuttgart, Stuttgart, Germany; Clinic for Thoracic and Cardiovascular Surgery, Herz-und Diabeteszentrum Bad Oeynhausen, Bad Oeynhausen, Germany; Department of Thoracic and Cardiovascular Surgery, University Hospital Schleswig-Holstein, Lübeck, Germany; Department of Medicine, University Medical Center Schleswig-Holstein, Lübeck, Germany; Medizinische Klinik I, St.-Johannes-Hospital Dortmund, Dortmund, Germany; Department of Cardiac Surgery, Goethe University Hospital, Frankfurt, Germany; Sana Klinikum Offenbach, Department of Cardiology, Offenbach, Germany

**Keywords:** surgical aortic valve replacement, SAVR, transcatheter valve implantation, TAVI, long-term outcomes, lung disease, patient-reported outcomes

## Abstract

**Objectives:**

Data from the German Aortic Valve Registry (GARY) were analysed to determine whether there are differences between patients with and without chronic lung disease (CLD) undergoing transcatheter aortic valve implantation (TAVI) and surgical aortic valve replacement (SAVR) and whether TAVI or SAVR is more beneficial in patients with preexisting CLD.

**Methods:**

Follow-up data from GARY registry patients treated from 2014 to 2015 and from 2018 to 2019 were incorporated in the analysis. Demographic results for each of the 2 treatment modalities were evaluated, and patients with and without CLD were compared. In a second step, variables that would have influenced the treatment decision in CLD patients in direction of either SAVR or TAVI were accounted for in the adjusted analysis. This led to a subgroup of 1385 patients with CLD that was subjected to propensity score weighted analysis to compare outcomes of TAVI and SAVR.

**Results:**

After exclusion, 11 457 SAVR patients and 2378 TAVI patients were analysed. CLD patients were sicker than patients without CLD, although the observed 30-day mortality was lower than expected by 3 risk scores. Accordingly, long-term survival in CLD patients was lower in both treatment groups than in patients without CLD. Thirty-day mortality was similar in the 2 treatment groups (SAVR 2.3%, TAVI 3.8%, *P* = .964) in spite of a possible selection bias in favour of SAVR. During a 5-year follow-up, survival after TAVI (44.6%) was significantly lower than after SAVR (56.7%), *P* = .029.

**Conclusions:**

Patients with CLD undergoing SAVR or TAVI are generally at higher risk for complications or death after the procedure than patients without CLD. After 5 years, patient-reported outcomes were similar in TAVI and in SAVR patients.

## Graphical abstract

**Figure:**
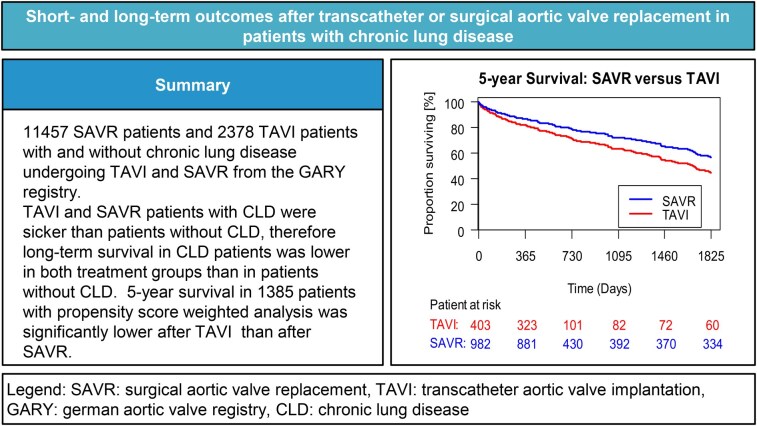

## INTRODUCTION

Patients with severe aortic stenosis are treated by transcatheter aortic valve implantation (TAVI) or by surgical aortic valve replacement (SAVR), mostly according to heart team decisions. Patients with preexisting pulmonary disease undergoing SAVR are at higher mortality risk than patients without.^1–4^ For TAVI patients, Crestanello *et al*^4^ found that in patients with chronic lung disease (CLD) from the Core Valve US pivotal trial, a long-term health benefit after TAVI was achieved only in selected patients. In fact, in TAVI patients with CLD, mortality at 1 and 3 years after the procedure was significantly higher than in patients without CLD.

The current European guidelines^5^ draw an arbitrary line at 75 years for allocating TAVI for older and SAVR for younger patients, whereas the American guidelines^6^ offer a broad age corridor between 65 and 80 years for treatment modality discussion. At the time of our patient inclusion, neither the current European guidelines^5^ nor the American guidelines,^6^ but the 2012 ESC/EACTS valve procedure guidelines^7^ were in place, which recommended that only patients with high risk for surgical replacement should undergo TAVI. Therefore, TAVI patients in our analysis were different from SAVR patients; we attempted to overcome these differences by propensity score adjusted analysis.

This analysis focused on 2 questions: first, whether patients with CLD admitted for aortic valve procedures have different preoperative conditions and demographics compared with patients without CLD, and, if so, whether this translates into different outcomes between the 2 aortic valve treatment modalities. Second, this study sought to determine whether TAVI or SAVR should be preferred in patients with preexisting lung disease. This issue has already been investigated by Dvir *et al*,^3^ who showed that in a cohort of patients from the randomized PARTNER 1 trial, the results of TAVI or SAVR in patients with preexisting lung disease were similar at the 1-year follow-up; however, the present study examined this question 5 years after the procedure. Randomized studies have shown that TAVI in patients with increased surgical risk results in equal^8^ or even better^9^ short-term mortality, whereas registries^10^ report worse long-term results at 5 years for TAVI patients.

## PATIENTS AND METHODS

### Definition of lung disease in GARY

The definition of lung disease in GARY comprises 5 items, according to the German Quality in Cardiac Surgery Program in 2014: 0 = no lung disease, 1 = yes, chronic obstructive pulmonary disease (CLD) with permanent medication, 2 = yes, CLD without permanent medication, 8 = yes, other lung diseases (defined as functional significant inflammatory and non-inflammatory lung and pleural diseases as well as tracheal stenoses), 9 = unknown. Combinations of CLD and other lung diseases had to be coded as 1 or 2. For this analysis, only patients with a “yes” score for items 1, 2, and 8 were classified as having chronic lung disease. No further specifications such as stages of disease or lung function parameters were available.^11^ For further definitions of GARY, please go to https://aortenklappenregister.de/downloads.html, and then to aolklreg 2020 sr 1 spec xml deutsches aortenklappenregister.zip and open “aoklreg_2019_sr0_ausfuellhinweise.”

### Patient selection for comparison of CLD vs no CLD in SAVR and TAVI arms

Between the years 2011 and 2019, 141 790 patients were included in the GARY registry, of which only 52 267 patients from 2014 to 2015 with a completed 5-year follow-up and from 2018 to 2019 with a 1-year follow-up were incorporated in the further analysis (**Figure S1**). Between 2014 and 2015, 93 hospitals entered patients into the GARY registry, between 2018 and 2019, 77 hospitals. In an attempt to create similar patient groups in the 2 treatment arms, factors were excluded that would bias the treatment decision in the direction of either SAVR or TAVI: patients with combined aortic valve replacement and coronary artery bypass grafting were excluded, and only those with solely treated by SAVR were included; frail patients, patients with limited prognosis, those with malignant disease, or those with porcelain aorta were excluded to limit our selection bias in favour of TAVI. Patients who underwent combined TAVI and PCI were also excluded as were patients with aortic aneurysms, because this skewed the decision towards SAVR (**Figure S1**).

### Patient selection for comparison of SAVR vs TAVI in CLD

After the first set of exclusions (see section above and **Figure S1**), a population of 2769 patients remained with acute or chronic lung disease. Further patients were excluded with left main stenosis and those with emergency indications because this would again bias treatment decision towards SAVR. We excluded concomitant coronary artery bypass grafting (CABG) procedures, because we wanted to analyse isolated SAVR and not the combination procedures. Combination procedures have a higher mortality than SAVR alone, so the results would be skewed in favour of TAVI (as often seen in the randomized studies). Patients with concomitant coronary artery disease (CAD), however, were left in the analysis, because this was not a risk-enhancing parameter.

Eight hundred and eight patients were excluded with OPS codes that indicated surgical procedures in addition to SAVR, and excluding patients ≥81 years of age (due to bias in favour of TAVI), 1385 patients remained for further analysis (**Figure S1**). In spite of all those efforts trying to minimize bias, we were not completely successful: The estimated risk scores before treatment all show a higher risk in the TAVI group.

### Statistical analysis

Variables with continuous values are described as mean ± SD, and categorical variables are described as frequencies and percentages. A weighted propensity score model including the variables age, sex, left ventricular ejection fraction, previous cardiac surgery, neurological dysfunction, renal replacement therapy, STS score, previous cardiac decompensation, previous PCI, mitral regurgitation, diabetes, pulmonary hypertension, atrial fibrillation, and peripheral arterial vascular disease was used for the non-randomized comparison of the treatment groups. SAVR patients were weighted to estimate outcomes for SAVR in a population comparable to the TAVI population (estimand average effect of treatment on the treated (ATT) with TAVI characterized as treated and SAVR as control) using gradient boosted logistic regression for calculating propensity score weights. As a sensitivity analysis, also a propensity score matched analysis (nearest neighbour method with caliper 0.1) using the same variables is reported in the supporting material (**Tables S3–S5**). Weighted and unweighted comparisons for continuous variables were performed by a non-parametric weighted or standard Wilcoxon Mann-Whitney test. Categorical variables were compared using standard and weighted univariate logistic regression with the Firth approach accounting for potentially small numbers in one group. Cox proportional hazard models were evaluated for all patients and for adjusted and unadjusted comparisons of SAVR vs TAVI cohorts using the end-points of 30-day, 1-year, and 5-year survival. Here, 5-year survival serves as the main end-point, 30-day and 1-year as key secondary end-points which are adjusted using Benjamini-Hochberg significance correction. The proportional hazard assumption was tested in all cases, and no significant deviations were observed. Log rank test and Kaplan-Meier curves were evaluated for all patients with SAVR and TAVI, respectively, to compare 30-day, 1-year, and 5-year survival between CLD vs no CLD groups. Again, the 30-day and 1-year survival as 2 key secondary end-points were adjusted using Benjamini-Hochberg significance correction. Furthermore, 5-year observational end-points in surviving patients are evaluated with a multiple imputation approach (imputation of multivariate missing data of the 5-year follow-up variables reported here using the propensity score variables for modelling and default settings of the R mice package) as there are many missing values in these variables. Statistical analysis was performed with SAS statistical software (version 9.4, SAS Institute, Cary, NC, USA) and R (R Foundation for Statistical Computing, Vienna, Austria) using, especially, the twang, survey, mice and EValues packages. *P* < .05 were considered statistically significant. In addition, E-values are calculated which represent the extent to which unmeasured confounders have to reach to nullify the observed hazard ratios. As the analyses were exploratory in nature, there was no further significance adjustment for multiple comparisons.

## RESULTS

### Comparison of CLD vs no CLD in SAVR and TAVI arms

**Figure 1.**
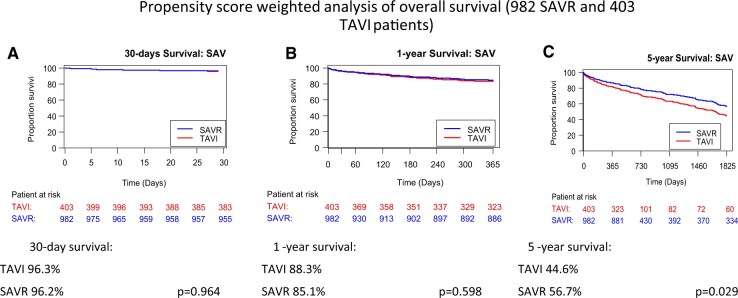
Propensity Score Weighted Analysis of Overall Survival (982 SAVR and 403 TAVI Patients). Survival rates after 30 days (A) and after 1 year (B) were not different, but were found to be lower in the TAVI group after 5 years (C)

**Figure 2.**
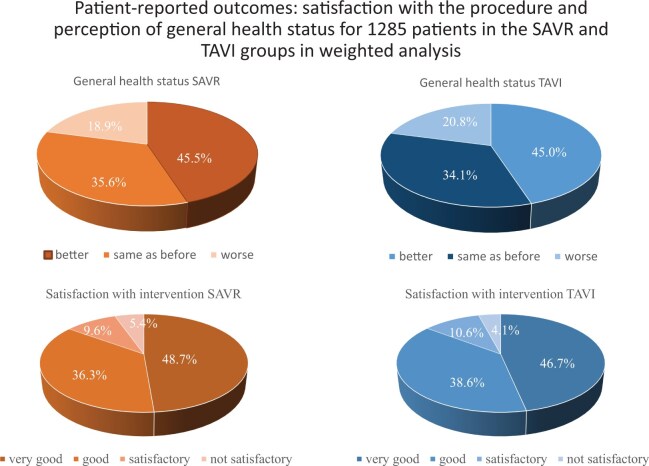
Patient-Reported Outcomes: Satisfaction With the Procedure and Perception of General Health Status for 1285 Patients in the TAVI and SAVR Groups in Weighted Analysis

In the first step of our analysis, patients with lung disease and patients without lung disease were compared for each of the 2 treatment modalities (final population A, **Figure S1**). The demographic data were different between the 2 treatment groups: in the SAVR group (*n* = 11 457), CLD patients were generally sicker than their counterparts, whereas in the TAVI group (*n* = 2378), CLD patients were younger but had more frequently hypertension, peripheral vascular disease, and permanent pacemakers than patients without CLD (**Tables S1 and S2**). In general, CLD patients were sicker, which was expressed by 3 risk scores that were higher for CLD patients. After either TAVI or SAVR, CLD patients stayed longer in the ICU than patients without CLD. In SAVR patients, new-onset atrial fibrillation was more frequent in those with CLD. In accordance with their poorer baseline conditions, CLD patients had worse short- and long-term survival in both treatment groups than patients without CLD: 1-year mortality in SAVR patients with CLD (7.8%) was higher than that without CLD (4.5%, *P* < .001), as was true for TAVI patients (CLD 16.9%, without CLD 10.6%, *P* = .001). Five-year survival in SAVR patients (**Figure 1**, **Figure S4**) was 71.6% with CLD and 83.2% without CLD (*P* < .001), and in TAVI patients, it was 43.6% with CLD and 57.2% without CLD (*P* < .001). Patient satisfaction with the procedure and the perception of general health status (**Figure 2**) was similar in SAVR patients with and without CLD, as was in TAVI patients; only 54.3% of CLD patients ranked the procedure as “very good,” whereas 56.9% of the patients without CLD ranked it as ‘very good’ (*P* = .424).

In the second step of our analysis, after further patient selection, the 2 operative techniques were compared in 1385 CLD patients: 982 in the SAVR group and 403 in the TAVI group (final population B, **Figure S1**). The comparability improved in the propensity score adjusted analysis (see **Figure S2** for standardized mean differences); nevertheless, there were still significant differences between unweighted SAVR (*n* = 982) and TAVI (*n* = 403) groups as shown in **Table 1**. Therefore, we also report results from a matched analysis (see **Figure S3** and **Table S3**) with overall comparable results.

**Table 1.** Periprocedural Details of 1395 Patients with Chronic Lung Disease from the GARY Registry

	SAVR weighted	TAVI	*P*-value
Age, years	75.265 (0.184)	75.774 (0.359)	.036
Sex			
Male	(61.5%)	236 (58.6%)	.438
Female	(38.5%)	167 (41.4%)	.438
BMI, kg/m²	29.639 (0.257)	29.143 (0.298)	.145
BMI classes, kg/m²			
<18.5	(3.2%)	7 (1.8%)	.228
18.5–25.0	(18.9%)	96 (24.3%)	.092
>25.0	(77.9%)	292 (73.9%)	.234
Creatinine	1.175 (0.020)	1.176 (0.021)	.983
NYHA			
I	(1.6%)	5 (1.2%)	.660
II	(18.5%)	44 (10.9%)	.005
III	(69.3%)	293 (72.7%)	.323
IV	(10.5%)	61 (15.1%)	.076
Status post-myocardial infarction	(8.9%)	70 (17.4%)	.002
Status post-PCI	(18.1%)	91 (22.6%)	.149
Permanent pacemaker	(12.7%)	49 (12.4%)	.921
Atrial fibrillation	(29.1%)	118 (29.3%)	.969
Mitral regurgitation			
<2°	(81.4%)	291 (72.2%)	.005
≥2°	(18.6%)	112 (27.8%)	.005
Tricuspid valve insufficiency			
None	(48.1%)	113 (28.5%)	<.001
Low	(43.3%)	218 (54.9%)	.003
Medium	(7.7%)	60 (15.1%)	.003
Severe	(0.9%)	6 (1.5%)	.491
Previous cardiac surgery	(15.5%)	83 (20.6%)	.084
Prior balloon valvuloplasty	(0.1%)	10 (2.5%)	.100
LVEF, %	53.547 (0.589)	51.406 (0.675)	.006
Hypertension	(87.8%)	362 (90.7%)	.220

	SAVR weighted	TAVI	*P*-value
Status postcardiac decompensation	(35.0%)	169 (41.9%)	.061
Cardiogenic shock			
None	(72.4%)	264 (65.7%)	.059
Yes, <48 hours	(2.4%)	15 (3.7%)	.331
Yes, <21 days	(13.2%)	63 (15.7%)	.363
Yes, >21 days	(12.0%)	60 (14.9%)	.262
Aortic valve opening area, cm^2^	0.783 (0.020)	0.799 (0.019)	.532
Mean aortic valve gradient, mmHg	41.232 (0.720)	38.617 (0.833)	.005
Aortic valve calcification			
None	(7.4%)	11 (2.9%)	.008
Low	(4.5%)	21 (5.5%)	.558
Medium	(14.3%)	139 (36.4%)	<.001
High	(73.8%)	211 (55.2%)	<.001
Neurologic dysfunction	(8.0%)	56 (13.9%)	.016
Renal replacement therapy			
No	(96.4%)	383 (95.0%)	.376
Acute	(1.0%)	7 (1.7%)	.406
Chronic	(2.6%)	13 (3.2%)	.628
Pulmonary hypertension			
No or ≤55 mmHg	(86.9%)	330 (81.9%)	.073
Yes, >55	(13.1%)	73 (18.1%)	.073
Diabetes	(40.7%)	163 (40.4%)	.941
AKL score	4.402 (0.220)	5.487 (0.197)	<.001
Euroscore	15.424 (0.561)	20.9 (0.529)	<.001
STS score	5.560 (0.207)	6.259 (0.196)	.011
Urgent indication	(19.5%)	48 (11.9%)	.006
Peripheral arterial vascular disease	(17.8%)	102 (25.3%)	.019

Propensity score weighting was used to compare the 982 patients in the SAVR group with the 403 patients in the TAVI group.

Abbreviations: AKL, German aortic valve score; BMI, body-mass index; LVEF, left ventricular ejection fraction; NYHA, New York Heart Association; PCI, percutaneous coronary intervention; SAVR; surgical aortic valve replacement; STS, Society of Thoracic Surgeons; TAVI, transcatheter aortic valve implantation.

In propensity score weighted analysis in the population with CLD, most demographic parameters were comparable except for the following: in the TAVI group, neurological dysfunction was more pronounced (13.9% vs SAVR 8.2%, *P* = .015), and peripheral arterial disease was more frequent (25.3% vs SAVR 17.9%, *P* = .018). These inter-group differences led to significant discrepancies in all 3 calculated risk scores: the STS score (TAVI 6.3 vs SAVR 5.6%, *P* = .048), the Euro score II (TAVI 6.9% vs SAVR 5.9%, *P* = .040), and the German aortic valve score (TAVI 5.5% vs SAVR 4.4%, *P* < .001) all predicted a higher short-term mortality of TAVI patients compared with SAVR patients (**Table 1**). In contrast, the observed 30-day mortality was similar in the 2 groups (SAVR 2.3%, TAVI 3.8%), as was 1-year mortality (SAVR 14.9%, TAVI 16.7%) (**Figure 1A and B**).

The duration of the procedures and the postoperative ICU stay between the groups in propensity score weighted analysis (**Table 2**) was longer for SAVR (177 min and 4.4 days) than for TAVI (77 min and 2.7 days) (*P* < .001 for both comparisons). Procedural complications such as vascular complications (SAVR 0.5%, TAVI 6.2%) and aortic regurgitation >2° (SAVR 0.8%, TAVI 3.4%) were more frequent in the TAVI group, whereas postimplantation valve gradients were lower after TAVI (**Table 2**).

**Table 2.** Early Postprocedural Outcomes Comparing 982 Patients in Weighted Analysis With 403 Patients in the TAVI Group

	SAVR weighted	TAVI	*P*-value
Stroke			
Yes	(2.5%)	6 (1.5%)	0.344
TIA			
Yes	(1.8%)	2 (0.5%)	0.115
Myocardial infarction			
Yes	(0.2%)	0 (0.0%)	0.997
New-onset AF			
Yes	(21.0%)	99 (25.1%)	0.215
New-onset pacer			
Yes	(4.9%)	23 (9.5%)	0.075
Bleeding			
<2 RBC units	(13.1%)	16 (22.5%)	0.080
≥2 RBC units	(86.9%)	55 (77.5%)	0.080
Vascular complication			
Yes	(0.4%)	25 (6.2%)	0.003
Postimplant mean gradient			
<10 mm Hg	(41.6%)	176 (55.3%)	0.003
10–14 mm Hg	(29.9%)	81 (25.5%)	0.280
≥15 mm Hg	(28.5%)	61 (19.2%)	0.015
New-onset dialysis			
Temporary	(4.0%)	8 (2.9%)	0.504
Chronic	(3.7%)	3 (1.1%)	0.065
Postprocedural stay in ICU, days	4.418 (0.213)	2.689 (0.251)	<0.001
Aortic incompetence			
None	(93.5%)	254 (65.8%)	<0.001
Grade I	(5.9%)	119 (30.8%)	<0.001
≥Grade II	(0.6%)	13 (3.4%)	0.033

Abbreviations: AF, atrial fibrillation; ICU, intensive care unit; RBC, red blood cells; SAVR, surgical aortic valve replacement; TAVI, transcatheter aortic valve implantation; TIA, transient ischaemic attack.

During a 5-year follow-up of the selected patients with CLD, mortality after TAVI (55.4%) was higher than after SAVR (43.3%) in weighted analysis (**Figure 1C**). This is also confirmed in the alternative propensity score matching adjustment (**Figure S4**). Evalue for the hazard ratio is 1.87, confirming that potential unobserved confounders have to have a high impact to eliminate this effect. All other long-term outcomes, such as rehospitalization or NYHA class, were similar between the 2 groups (**Table 3**).

**Table 3.** Late Outcomes During a 5-Year Follow-Up of 1395 Patients With Chronic Lung Disease From the GARY Registry, Comparing 982 Patients in the SAVR Group in Weighted Analysis With 403 Patients in the TAVI Group

	SAVR weighted	TAVI	*P*-value
Myocardial infarction	(1.1%)	1 (0.6%)	.6177
New pacemaker/ICD implant	(7.0%)	29 (13.4%)	.044
Stroke	(3.4%)	5 (2.0%)	.365
TIA	(1.1%)	4 (1.6%)	.653
Coronary artery bypass surgery	(0.1%)	1 (0.4%)	.527
PCI/balloon dilatation	(0.7%)	3 (1.2%)	.600
Further hospitalization	(41.9%)	106 (42.6%)	.888
Further hospitalization due to complications related to the aortic valve intervention	(17.5%)	10 (9.9%)	.130
Further hospitalization due to heart or circulatory problems	(43.0%)	39 (38.2%)	.504
NYHA			
I	(32.8%)	72 (29.3%)	.417
II	(35.1%)	77 (31.3%)	.383
III	(24.6%)	82 (33.3%)	.040
IV	(7.5%)	15 (6.1%)	.547

Abbreviations: ICD, implantable cardioverter defibrillator; NYHA, New York Heart Association; PCI, percutaneous coronary intervention; SAVR; surgical aortic valve replacement; TAVI, transcatheter aortic valve implantation, TIA, transient ischaemic attack.

The valve platforms used (**Table S6**) were mainly Edwards Sapien, Medtronic Core valve, and Symetis/Boston Scientific developments for TAVI, and Edwards Perimount, Medtronic Hancock, and Abbott (SJM Trifecta) valves for biological SAVR. In the whole SAVR group, from the 11 457 aortic valve procedures, 1230 (10.7%) were mechanical valves.

The patient-reported outcome survey compared the patients’ general health status 5 years after the procedure with their health status before TAVI or SAVR, which was similar between the 2 groups in weighted analysis: about 45% reported their health to be better, and only 19–20% reported it to be worse after 5 years (*P* > .200) (**Figure 2**). The patients’ satisfaction with the valve procedure was also queried: similar results (*P* > .200) were reported after TAVI and SAVR, with the majority of the patients reporting a very high satisfaction and only 2.4% (TAVI) and 3.6% (SAVR) claiming the result to be unsatisfactory after 5 years.

## DISCUSSION

Our retrospective, propensity score adjusted analysis of the GARY data from 2014 to 2015 and 2018 to 2019 yielded several results: (1) independent of the type of valve procedure, CLD patients have a higher procedural risk profile and a higher long-term mortality than patients without CLD; (2) among CLD patients, the short-term outcomes in both groups were better than predicted by contemporary risk scores; (3) there was no difference in short-term mortality after SAVR or TAVI in CLD patients, although side effects such as vascular complications and aortic regurgitation occurred more often in the TAVI group; (4) during long-term follow-up, TAVI patients had a higher 5-year mortality than their SAVR counterparts.

### CLD risk profile

For our analysis, 3 different risk scores were applied: the Euroscore II, the STS score, and the German aortic valve score (AKL). The AKL score was developed in Germany^12^ and has been validated for both SAVR and TAVI procedures. CLD is a known risk factor after SAVR and TAVI^1–4^ and is therefore part of the risk estimation of all 3 scores. After either SAVR or TAVI, 30-day mortality was higher in patients with CLD than in patients without CLD. This is not a new finding, and this result confirms the findings reported by Dvir *et al*^3^ and Crestanello *et al.*^4^ Interestingly, the mortality was overestimated by all 3 scores, not just the Euroscore. In contrast, De Miguel-Diez *et al*^13^ found that for SAVR patients, no differences were found for early mortality between patients with and without CLD. Their conclusion was that CLD *per se* should not represent a contraindication for SAVR. The results of the present study confirm this finding.

### Short-term results after TAVI or SAVR in CLD patients

As mentioned above, 30-day mortality in our registry patients was low, with no significant difference between groups (TAVI 3.8%, SAVR 2.3%), and it was even lower than expected. The commonly reported vascular complications were different in the 2 groups, which is understandable given the need for femoral vascular access in TAVI patients. This difference was also reported by Adams *et al*^9^ as well as by Leon *et al.*^8^ The higher bleeding rate and transfusion amount after SAVR is also known and is certainly a dilution effect during the use of the heart-lung machine as well as a sequela of open-heart surgery; this has also been reported by Adams *et al*^9^ and Dvir *et al.*^3^

It is known from several reports^8^^,^^14^ that the periprocedural pacemaker implantation rate is higher in TAVI patients. Accordingly, our data show that SAVR patients (4.9%) had nearly half as many early postoperative pacemaker implants as TAVI patients (9.5%), but this did not reach statistical significance (**Table 2**). Moreover, in our registry, we found—similar to Abdelgawad *et al.*^14^—that new-onset atrial fibrillation occurred similarly often after TAVI and SAVR (**Table 2**).

### Long-term results after TAVI or SAVR in CLD patients

In 2014, Dvir *et al*^3^ published the results of the PARTNER 1 trial for patients with CLD. They showed that the 2-year mortality of TAVI and SAVR patients (35.2% vs 33.6%) was similar, as observed in the 1-year registry results of the present study: the mortality of TAVI patients was similar to that of SAVR patients. In contrast, 5-year mortality was higher in TAVI patients. Disappointingly, no follow-up data from the randomized PARTNER 1 cohort for those patients with CLD are available in order to compare the PARTNER 1 results with those from our registry.

Our results tend to reflect those of the GARY registry as a whole,^10^ but they contradict the 5-year results of the PARTNER 2 trial.^15^ The GARY 5-year data are based on 2 well-balanced cohorts, which were matched and analysed retrospectively, whereas our patient groups differ in several parameters (**Table 1**) despite a thorough propensity score adjustment. There is an ongoing discussion regarding the value of registries compared to randomized studies. We think that our study is an important contribution to this discussion, because it shows that bias in registries is unavoidable and that the exactness of an indication process can influence the registry’s results. Randomized studies include only small, selected patient groups and therefore the results are not generalizable.

Patient-reported outcomes in our study were not based on standard quality-of-life questionnaires but were rather taken from the patients’ perception of the procedure after 5 years. In accordance with the results of Baron *et al*,^16^ who found no differences between TAVI and SAVR patients in the patient-reported outcomes after 2 years in the PARTNER 2-cohort, there were no differences regarding patient satisfaction and general health status in the current study. In this context, the findings of Crestanello *et al*^4^ are very thought provoking: the authors described that in their study cohort of TAVI patients with CLD, only 43% had a favourable health benefit at 1 year and only 22.5% at 3 years. Crestanello *et al*^4^ defined health benefit as (1) alive, (2) quality-of-life score corresponding to NYHA I–II, and (3) stability or improvement in the measured quality-of-life score (KCCQ). Although the NYHA class improved in >80% of their patients and quality-of-life scores improved by 20 points, the combined end-point for health benefit was quite disappointing reached by less than half of the patients.

### Limitations

Although our patients underwent a thorough propensity score adjustment process to reduce the inherent bias in favour of one or the other procedure, the possibility that a bias existed cannot be excluded: first, there are factors we were possibly not aware of that were not taken into account, and second, the selection process of patients for TAVI or SAVR was subject to the 2012 ESC/EACTS guidelines^7^ that scheduled only high-risk patients or patients with contraindications for SAVR as TAVI candidates.

Only in 2016, the intermediate-term results of the PARTNER 2 trial^8^ were published, and the results of the PARTNER 3 trial were not available. Therefore, it was clear that GARY patients with a high and intermediate perioperative risk were allocated to the TAVI group, whereas the majority of low-risk patients went into the SAVR group. Our results (**Table S1**) for the unadjusted groups show exactly this distribution pattern.

The definition of lung disease for the German Quality Assessment and for GARY was similar and did not account for the severity of the disease or for the stages of chronic obstructive pulmonary disease or other lung dysfunctions as this was not within the scope of the registry. Therefore, exact measurements of lung function parameters were not available, although Pino *et al*^17^ showed that CLD, when correctly classified, does not predict clinically relevant postoperative outcomes. Moreover, the lung disease definitions of GARY and of the Society of Thoracic Surgeons are different, making comparisons of patients with CLD between these 2 groups nearly impossible.

## CONCLUSIONS

Patients with lung disease undergoing SAVR or TAVI generally are at higher risk for complications or death after the procedure than patients without lung disease. In patients with CLD who underwent SAVR or TAVI, it was difficult to adjust for baseline parameters. Although periprocedural mortality was similar, a higher 5-year mortality of CLD patients was observed in the TAVI group, possibly due to a higher predicted periprocedural risk in the TAVI group even in propensity score weighted analysis.

## Supplementary Material

ivaf189_Supplementary_Data

## Data Availability

The data underlying this article were provided by the GARY registry executive board by permission. Data will be shared on request to the corresponding author with permission of the executive board.
