# Supplementary material for: Short- and Long-Term Outcomes After Transcatheter or Surgical Aortic Valve Replacement in Patients With Chronic Lung Disease: An Analysis From the German Aortic Valve Registry
Source: Interdiscip Cardiovasc Thorac Surg. 2025 Aug 18;40(8):ivaf189. doi: 10.1093/icvts/ivaf189 (PMC12396625; doi:10.1093/icvts/ivaf189)
Supplement: ivaf189_Supplementary_Data [file ivaf189_supplementary_data.zip › Supplementary Tab. 1-6.docx]

Suppl. Table 1: Demographic data of 11,457 patients with and without chronic lung disease from the GARY registry in the SAVR group

| SAVR |  |  |  |  |
| --- | --- | --- | --- | --- |
|  | Missings | COPD | no COPD | p-value |
| Age, years | 0.0% | 69.6 (0.313) | 68.8 (0.096) | 0.669 |
| Gender |  |  |  |  |
| male | 0.0% | 634 (64.6%) | 6271 (59.9%) | 0.004 |
| female | 0.0% | 348 (35.4%) | 4204 (40.1%) | 0.004 |
| BMI, kg/m² | 0.5% | 29.8 (0.793) | 28.8 (0.243) | <0.001 |
| BMI classes |  |  |  |  |
| < 18.5 kg/m² | 0.7% | 12 (1.2%) | 45 (0.4%) | 0.002 |
| 18.5 to 25.0 kg/m² | 0.7% | 189 (19.4%) | 2613 (25.1%) | <0.001 |
| > 25.0 kg/m² | 0.7% | 775 (79.4%) | 7739 (74.4%) | <0.001 |
| Creatinine | 1.0% | 1.042 (0.010) | 0.978 (0.003) | <0.001 |
| NYHA |  |  |  |  |
| I | 0.0% | 25 (2.5%) | 669 (6.4%) | <0.001 |
| II | 0.0% | 248 (25.3%) | 3992 (38.1%) | <0.001 |
| III | 0.0% | 644 (65.6%) | 5461 (52.1%) | <0.001 |
| IV | 0.0% | 65 (6.6%) | 353 (3.4%) | <0.001 |
| Status post-myocardial infarction | 0.2% | 58 (5.9%) | 452 (4.3%) | 0.024 |
| Status post-PCI | 0.0% | 91 (9.3%) | 847 (8.1%) | 0.191 |
| Permanent pacemaker | 0.0% | 55 (5.6%) | 331 (3.2%) | <0.001 |
| Atrial fibrillation | 0.0% | 157 (16.0%) | 1136 (10.8%) | <0.001 |
| Mitral regurgitation |  |  |  |  |
| <2° | 3.4% | 911 (92.8%) | 9328 (92.5%) | 0.779 |
| ≥2° | 3.4% | 71 (7.2%) | 758 (7.5%) | 0.779 |
| Aortic regurgitation |  |  |  |  |
| no or minor | 2.3% | 734 (75.7%) | 7622 (74.6%) | 0.434 |
| moderate or severe | 2.3% | 235 (24.3%) | 2597 (25.4%) | 0.434 |
| Tricuspid valve insufficiency |  |  |  |  |
| none | 4.7% | 603 (62.4%) | 6013 (60.4%) | 0.220 |
| low | 4.7% | 324 (33.5%) | 3633 (36.5%) | 0.068 |
| medium | 4.7% | 34 (3.5%) | 282 (2.8%) | 0.210 |
| severe | 4.7% | 5 (0.5%) | 28 (0.3%) | 0.172 |
| Previous cardiac surgery | 0.1% | 75 (7.6%) | 659 (6.3%) | 0.100 |
| Prior balloon valvuloplasty | 2.8% | 4 (0.4%) | 40 (0.4%) | 0.762 |
| LVEF, % | 13.0% | 55.4 (0.391) | 57.4 (0.118) | <0.001 |
| Hypertension | 1.5% | 832 (85.2%) | 8291 (80.4%) | <0.001 |
| Status post-cardiac decompensation | 0.0% | 161 (16.4%) | 1020 (9.7%) | <0.001 |
| Cardiogenic shock |  |  |  |  |
| none | 0.3% | 835 (85.3%) | 9698 (92.8%) | <0.001 |
| yes,<48 hours | 0.3% | 13 (1.3%) | 50 (0.5%) | 0.002 |
| yes,<21 days | 0.3% | 64 (6.5%) | 371 (3.6%) | <0.001 |
| yes,>21 days | 0.3% | 67 (6.8%) | 329 (3.1%) | <0.001 |
| Aortic valve orifice area, cm² | 21.6% | 0.794 (0.011) | 0.788 (0.003) | 0.053 |
| Mean aortic valve gradient, mmHg | 21.7% | 45.2 (0.616) | 46.9 (0.191) | 0.003 |
| Aortic valve calcification |  |  |  |  |
| none | 5.0% | 79 (8.2%) | 683 (6.9%) | 0.123 |
| low | 5.0% | 47 (4.9%) | 376 (3.8%) | 0.094 |
| medium | 5.0% | 138 (14.3%) | 1699 (17.1%) | 0.026 |
| high | 5.0% | 699 (72.6%) | 7163 (72.2%) | 0.809 |
| Neurologic dysfunction | 0.1% | 82 (8.4%) | 700 (6.7%) | 0.050 |
| Renal replacement therapy |  |  |  |  |
| no | 0.0% | 968 (98.6%) | 10383 (99.1%) | 0.086 |
| acute | 0.0% | 4 (0.4%) | 14 (0.1%) | 0.048 |
| chronic | 0.0% | 10 (1.0%) | 78 (0.7%) | 0.300 |
| Pulmonary hypertension |  |  |  |  |
| no or ≤55mmHg | 1.8% | 935 (95.2%) | 9988 (97.2%) | <0.001 |
| yes, >55 | 1.8% | 47 (4.8%) | 283 (2.8%) | <0.001 |
| Diabetes | 0.1% | 302 (30.8%) | 2487 (23.8%) | <0.001 |
| Peripheral arterial vascular disease | 0.0% | 81 (8.2%) | 382 (3.6%) | <0.001 |
| AKL score | 2.8% | 2.466 (0.051) | 1.522 (0.016) | <0.001 |
| Euroscore | 2.7% | 3.459 (0.101) | 2.422 (0.031) | <0.001 |
| STS score | 0.0% | 3.461 (0.053) | 1.976 (0.016) | <0.001 |
| Urgent indication | 0.0% | 128 (13.0%) | 995 (9.5%) | <0.001 |
| Open sternotomy | 0.0% | 712 (72.5%) | 7287 (69.6%) | 0.055 |
| Biological valve | 0.0% | 887 (90.3%) | 9319 (89.0%) | 0.210 |
| Valve diameter, mm | 0.1% | 23.5 (0.088) | 23.6 (0.027) | 0.963 |

Abbreviations: COPD, chronic obstructive pulmonary disease; BMI, body-mass index; NYHA, New York Heart Association; TAVI, transcatheter aortic valve implantation, SAVR; surgical aortic valve replacement; PCI, percutaneous coronary intervention; LVEF, left ventricular ejection fraction; AKL, German aortic valve score; STS, Society of Thoracic Surgeons

The valve diameter reflects the labeled valve size of the manufacturer.

Suppl. Table 2: Demographic data of 2,378 patients with and without chronic lung disease from the GARY registry in the TAVI group

| TAVI |  |  |  |  |
| --- | --- | --- | --- | --- |
|  | Missings | COPD | no COPD | p-value |
| Age, years | 0.0% | 75.8 (0.218) | 76.4 (0.099) | 0.001 |
| Gender |  |  |  |  |
| male | 0.0% | 236 (58.6%) | 1076 (54.5%) | 0.134 |
| female | 0.0% | 167 (41.4%) | 899 (45.5%) | 0.134 |
| BMI, kg/m² | 1.1% | 29.1 (10.301) | 33.8 (4.656) | 0.085 |
| BMI classes |  |  |  |  |
| < 18.5 kg/m² | 1.6% | 7 (1.8%) | 17 (0.9%) | 0.105 |
| 18.5 to 25.0 kg/m² | 1.6% | 96 (24.3%) | 526 (27.0%) | 0.266 |
| > 25.0 kg/m² | 1.6% | 292 (73.9%) | 1402 (72.1%) | 0.465 |
| Creatinine | 4.1% | 1.176 (0.025) | 1.135 (0.011) | 0.141 |
| NYHA |  |  |  |  |
| I | 0.0% | 5 (1.2%) | 58 (2.9%) | 0.050 |
| II | 0.0% | 44 (10.9%) | 337 (17.1%) | 0.002 |
| III | 0.0% | 293 (72.7%) | 1376 (69.7%) | 0.229 |
| IV | 0.0% | 61 (15.1%) | 204 (10.3%) | 0.006 |
| Status post-myocardial infarction | 0.1% | 70 (17.4%) | 282 (14.3%) | 0.112 |
| Status post-PCI | 0.0% | 91 (22.6%) | 487 (24.7%) | 0.385 |
| Permanent pacemaker | 0.0% | 49 (12.2%) | 155 (7.8%) | 0.006 |
| Atrial fibrillation | 0.0% | 118 (29.3%) | 523 (26.5%) | 0.245 |
| Mitral regurgitation |  |  |  |  |
| <2° | 2.9% | 291 (72.2%) | 1484 (77.9%) | 0.015 |
| ≥2° | 2.9% | 112 (27.8%) | 421 (22.1%) | 0.015 |
| Aortic regurgitation |  |  |  |  |
| no or minor | 2.7% | 321 (80.5%) | 1546 (80.7%) | 0.876 |
| moderate or severe | 2.7% | 78 (19.5%) | 369 (19.3%) | 0.876 |
| Tricuspid valve insufficiency |  |  |  |  |
| none | 4.9% | 113 (28.5%) | 567 (30.4%) | 0.452 |
| low | 4.9% | 218 (54.9%) | 1031 (55.3%) | 0.890 |
| medium | 4.9% | 60 (15.1%) | 213 (11.4%) | 0.042 |
| severe | 4.9% | 6 (1.5%) | 54 (2.9%) | 0.126 |
| Previous cardiac surgery | 0.1% | 83 (20.6%) | 524 (26.6%) | 0.012 |
| Prior balloon valvuloplasty | 2.6% | 10 (2.5%) | 34 (1.8%) | 0.298 |
| LVEF, % | 9.4% | 51.406 (0.699) | 52.037 (0.309) | 0.501 |
| Hypertension | 2.0% | 362 (90.7%) | 1673 (86.6%) | 0.022 |
| Status post-cardiac decompensation | 0.0% | 169 (41.9%) | 570 (28.9%) | <0.001 |
| Cardiogenic shock |  |  |  |  |
| none | 0.3% | 264 (65.7%) | 1543 (78.3%) | <0.001 |
| yes,<48 hours | 0.3% | 15 (3.7%) | 46 (2.3%) | 0.105 |
| yes,<21 days | 0.3% | 63 (15.7%) | 173 (8.8%) | <0.001 |
| yes,>21 days | 0.3% | 60 (14.9%) | 208 (10.6%) | 0.013 |
| Aortic valve orifice area, cm² | 8.8% | 0.777 (0.012) | 0.750 (0.005) | 0.031 |
| Mean aortic valve gradient, mmHg | 10.8% | 38.6 (0.797) | 41.4 (0.362) | <0.001 |
| Aortic valve calcification |  |  |  |  |
| none | 6.6% | 11 (2.9%) | 58 (3.2%) | 0.856 |
| low | 6.6% | 21 (5.5%) | 103 (5.6%) | 0.993 |
| medium | 6.6% | 139 (36.4%) | 631 (34.3%) | 0.433 |
| high | 6.6% | 211 (55.2%) | 1047 (56.9%) | 0.540 |
| Neurologic dysfunction | 0.1% | 56 (13.9%) | 281 (14.2%) | 0.885 |
| Renal replacement therapy |  |  |  |  |
| no | 0.0% | 383 (95.0%) | 1897 (96.1%) | 0.325 |
| acute | 0.0% | 7 (1.7%) | 9 (0.5%) | 0.009 |
| chronic | 0.0% | 13 (3.2%) | 69 (3.5%) | 0.860 |
| Pulmonary hypertension |  |  |  |  |
| no or ≤55mmHg | 1.6% | 330 (81.9%) | 1636 (84.4%) | 0.203 |
| yes, >55 | 1.6% | 73 (18.1%) | 302 (15.6%) | 0.203 |
| Diabetes | 0.0% | 163 (40.4%) | 714 (36.2%) | 0.105 |
| Peripheral arterial vascular disease | 0.0% | 102 (25.3%) | 301 (15.2%) | <0.001 |
| AKL score | 3.2% | 5.487 (0.211) | 3.793 (0.096) | <0.001 |
| Euroscore | 3.0% | 6.876 (0.333) | 5.747 (0.152) | <0.001 |
| STS score | 0.0% | 6.259 (0.177) | 3.909 (0.080) | <0.001 |
| Urgent indication | 0.0% | 48 (11.9%) | 214 (10.8%) | 0.506 |
| Biological valve | 0.0% | 403 (100.0%) | 1975 (100.0%) | 0.449 |
| Valve diameter, mm | 0.3% | 26.8 (0.218) | 26.7 (0.099) | 0.515 |
|  |  |  |  |  |
|  |  |  |  |  |

Abbreviations: COPD, chronic obstructive pulmonary disease; BMI, body-mass index; NYHA, New York Heart Association; TAVI, transcatheter aortic valve implantation, SAVR; surgical aortic valve replacement; PCI, percutaneous coronary intervention; LVEF, left ventricular ejection fraction; AKL, German aortic valve score; STS, Society of Thoracic Surgeons

The valve diameter reflects the labeled valve size of the manufacturer.

Suppl. Table 3: Demographic data of SAVR and TAVI patients with a propensity score matching

|  | Missings | SAVR | TAVI | p-value |
| --- | --- | --- | --- | --- |
| Age, years | 0.0% | 74.893 (0.267) | 75.049 (0.267) | 0.410 |
| Gender |  |  |  |  |
| male | 0.0% | 161 (59.0%) | 156 (57.1%) | 0.665 |
| female | 0.0% | 112 (41.0%) | 117 (42.9%) | 0.665 |
| BMI, kg/m² | 0.4% | 29.134 (0.364) | 29.494 (0.364) | 0.498 |
| BMI classes |  |  |  |  |
| < 18.5 kg/m² | 1.5% | 6 (2.2%) | 3 (1.1%) | 0.341 |
| 18.5 to 25.0 kg/m² | 1.5% | 58 (21.5%) | 62 (23.1%) | 0.646 |
| > 25.0 kg/m² | 1.5% | 206 (76.3%) | 203 (75.7%) | 0.881 |
| Creatinine | 2.9% | 1.080 (0.025) | 1.132 (0.025) | 0.304 |
| NYHA |  |  |  |  |
| I | 0.0% | 6 (2.2%) | 4 (1.5%) | 0.541 |
| II | 0.0% | 57 (20.9%) | 33 (12.1%) | 0.006 |
| III | 0.0% | 186 (68.1%) | 205 (75.1%) | 0.072 |
| IV | 0.0% | 24 (8.8%) | 31 (11.4%) | 0.323 |
| Status post-myocardial infarction | 0.2% | 20 (7.4%) | 42 (15.4%) | 0.003 |
| Status post-PCI | 0.0% | 54 (19.8%) | 44 (16.1%) | 0.267 |
| Permanent pacemaker | 0.0% | 28 (10.3%) | 36 (13.2%) | 0.290 |
| Atrial fibrillation | 0.0% | 74 (27.1%) | 70 (25.6%) | 0.698 |
| Mitral regurgitation |  |  |  |  |
| <2° | 0.0% | 224 (82.1%) | 220 (80.6%) | 0.662 |
| ≥2° | 0.0% | 49 (17.9%) | 53 (19.4%) | 0.662 |
| Aortic regurgitation |  |  |  |  |
| no or minor | 1.1% | 203 (75.5%) | 227 (83.8%) | 0.017 |
| moderate or severe | 1.1% | 66 (24.5%) | 44 (16.2%) | 0.017 |
| Tricuspid valve insufficiency |  |  |  |  |
| none | 1.6% | 132 (49.6%) | 84 (31.0%) | <0.001 |
| low | 1.6% | 113 (42.5%) | 152 (56.1%) | 0.002 |
| medium | 1.6% | 18 (6.8%) | 32 (11.8%) | 0.045 |
| severe | 1.6% | 3 (1.1%) | 3 (1.1%) | 0.980 |
| Previous cardiac surgery | 0.0% | 41 (15.0%) | 39 (14.3%) | 0.810 |
| Prior balloon valvuloplasty | 1.1% | 0 (0.0%) | 9 (3.3%) | 0.002 |
| LVEF, % | 12.8% | 52.924 (0.843) | 53.160 (0.843) | 0.698 |
| Hypertension | 0.7% | 243 (89.0%) | 247 (91.8%) | 0.270 |
| Status post-cardiac decompensation | 0.0% | 85 (31.1%) | 81 (29.7%) | 0.710 |
| Cardiogenic shock |  |  |  |  |
| none | 0.2% | 207 (76.1%) | 199 (72.9%) | 0.391 |
| yes,<48 hours | 0.2% | 7 (2.6%) | 5 (1.8%) | 0.569 |
| yes,<21 days | 0.2% | 30 (11.0%) | 34 (12.5%) | 0.608 |
| yes,>21 days | 0.2% | 28 (10.3%) | 35 (12.8%) | 0.359 |
| Aortic valve orifice area, cm² | 12.8% | 0.800 (0.019) | 0.774 (0.018) | 0.697 |
| Mean aortic valve gradient, mmHg | 14.7% | 42.253 (1.094) | 39.462 (1.022) | 0.043 |
| Aortic valve calcification |  |  |  |  |
| none | 3.1% | 21 (7.8%) | 6 (2.3%) | 0.004 |
| low | 3.1% | 16 (6.0%) | 17 (6.5%) | 0.797 |
| medium | 3.1% | 39 (14.6%) | 101 (38.7%) | <0.001 |
| high | 3.1% | 192 (71.6%) | 137 (52.5%) | <0.001 |
| Neurologic dysfunction | 0.0% | 28 (10.3%) | 33 (12.1%) | 0.500 |
| Renal replacement therapy |  |  |  |  |
| no | 0.0% | 267 (97.8%) | 263 (96.3%) | 0.322 |
| acute | 0.0% | 2 (0.7%) | 4 (1.5%) | 0.444 |
| chronic | 0.0% | 4 (1.5%) | 6 (2.2%) | 0.541 |
| Pulmonary hypertension |  |  |  |  |
| no or ≤55mmHg | 0.0% | 244 (89.4%) | 244 (89.4%) | 1.000 |
| yes, >55 | 0.0% | 29 (10.6%) | 29 (10.6%) | 1.000 |
| Diabetes | 0.0% | 108 (39.6%) | 104 (38.1%) | 0.726 |
| Peripheral arterial vascular disease | 0.0% | 42 (15.4%) | 47 (17.2%) | 0.564 |
| AKL score | 0.9% | 4.053 (0.256) | 4.212 (0.254) | 0.062 |
| Euroscore | 0.7% | 5.717 (0.379) | 5.031 (0.378) | 0.756 |
| STS score | 0.0% | 4.926 (0.218) | 5.140 (0.218) | 0.058 |
| Urgent indication | 0.0% | 48 (17.6%) | 35 (12.8%) | 0.123 |
| Open sternotomy | 0.0% | 209 (76.6%) | 0 (0.0%) | <0.001 |
| Biological valve | 0.0% | 268 (98.2%) | 273 (100.0%) | 0.027 |
| Valve diameter, mm | 0.0% | 23.308 (0.140) | 26.670 (0.140) | <0.001 |

Suppl. Table 4: Intra- and postoperative data of SAVR and TAVI patients with a propensity score matching

|  | Missings | SAVR | TAVI | p-value |
| --- | --- | --- | --- | --- |
| Procedural Duration | 0.0% | 179.3 (2.874) | 72.9 (2.874) | <0.001 |
| Stroke | 0.9% | 5 (1.8%) | 3 (1.1%) | 0.503 |
| TIA | 2.2% | 0 (0.0%) | 1 (0.4%) | 0.472 |
| Myocardial Infarction | 23.3% | 1 (0.4%) | 0 (0.0%) | 0.595 |
| New Onset Afib | 11.2% | 48 (19.4%) | 55 (23.1%) | 0.324 |
| New Onset Pacer | 36.6% | 8 (4.6%) | 15 (8.7%) | 0.136 |
| Bleeding |  |  |  |  |
| no RBC units | 1.6% | 136 (50.6%) | 230 (85.8%) | <0.001 |
| <2 RBC units | 1.6% | 17 (6.3%) | 11 (4.1%) | 0.255 |
| ≥2 RBC units | 1.6% | 116 (43.1%) | 27 (10.1%) | <0.001 |
| Vascular Complication | 0.0% | 1 (0.4%) | 13 (4.8%) | <0.001 |
| Post Implant Mean Gradient |  |  |  |  |
| <10mmHg | 28.4% | 65 (37.4%) | 117 (53.9%) | 0.001 |
| 10-14mmHg | 28.4% | 57 (32.8%) | 59 (27.2%) | 0.231 |
| ≥15mmHg | 28.4% | 52 (29.9%) | 41 (18.9%) | 0.012 |
| New Onset Dialysis |  |  |  |  |
| no | 23.3% | 226 (94.6%) | 176 (97.8%) | 0.105 |
| temporary | 23.3% | 8 (3.3%) | 3 (1.7%) | 0.317 |
| chronic | 23.3% | 5 (2.1%) | 1 (0.6%) | 0.227 |
| Post-OP stay in ICU (days) | 0.2% | 3.883 (0.308) | 2.548 (0.308) | <0.001 |
| Aortic Incompetence |  |  |  |  |
| none | 3.7% | 244 (93.1%) | 172 (65.2%) | <0.001 |
| grade I | 3.7% | 16 (6.1%) | 84 (31.8%) | <0.001 |
| ≥grade II | 3.7% | 2 (0.8%) | 8 (3.0%) | 0.062 |

Suppl. Table 5: Intra- and postoperative data of SAVR and TAVI patients with a propensity score matching with imputations

|  | SAVR | TAVI | p-value |
| --- | --- | --- | --- |
| Myokard Infarction | 4.7% | 4.0% | 0.835 |
| New Pacer ICD (excluding patients with pre-intervention pacer | 33.3% | 36.8% | 0.585 |
| Stroke | 37.7% | 38.8% | 0.872 |
| TIA | 12.0% | 13.7% | 0.746 |
| Bypass | 2.2% | 3.1% | 0.680 |
| PCI/Balloon Dilatation | 4.6% | 6.1% | 0.690 |
| Further Hospitalisation | 49.2% | 50.7% | 0.839 |
| Further Hospitalisation due to complications related to the aortic valve intervention | 8.1% | 6.4% | 0.737 |
| Further Hospitalisation due to heart or circulatory problems | 36.3% | 36.1% | 0.986 |
| NYHA |  |  |  |
| I | 32.5% | 36.3% | 0.540 |
| II | 37.1% | 37.7% | 0.912 |
| III | 27.1% | 23.1% | 0.412 |
| IV | 3.1% | 2.8% | 0.911 |
| Comparison of curent general health status to health status befor intervention |  |  |  |
| better | 44.4% | 45.5% | 0.903 |
| same as before | 36.3% | 32.4% | 0.618 |
| worse | 19.2% | 21.9% | 0.520 |
| Patient satisfaction with the intervantion |  |  |  |
| very good | 53.4% | 57.9% | 0.509 |
| good | 36.7% | 34.1% | 0.720 |
| satisfactory | 6.6% | 5.2% | 0.734 |
| not satisfactory | 3.1% | 2.0% | 0.716 |

Suppl. Table 6: Manufacturers and models of valve replacement

| TAVI |  | SAVR |  |
| --- | --- | --- | --- |
| Boston Scientific Acurate+ neo | 16 | Sorin Carbomedics | 78 |
| Symetis Acurate | 142 | Edwards Prima Plus Stentless | 31 |
| Edwards Sapien 3 | 790 | Carpentier-Edwards Bio | 55 |
| Edwards Sapien transapical | 231 | Edwards Perimount | 2879 |
| Edwards Sapien retroflex 3 | 79 | Edwards Magna + Magna Ease | 1943 |
| Edwards Sapien Novaflex | 93 | Edwards Intuity | 389 |
| Jena Valve | 32 | Labcor Valves | 79 |
| Medtronic Core Valve | 306 | On X Mechanical | 29 |
| Medtronic Engager Transpapical | 14 | Medtronic 3 f Bio | 40 |
| Medtronic Evolut + Pro | 384 | Medtronic mechanical | 245 |
|  |  | Medtronic Freestyle | 68 |
| other TAVI | 233 | Medtronic Hancock | 1088 |
|  |  | Medtronic Mosaic | 85 |
|  |  | Sorin Mitroflow | 266 |
|  |  | Sorin Bicarbon + other mechan. valves | 97 |
|  |  | Sorin Perceval | 546 |
|  |  | Sorin Stentless | 123 |
|  |  | Sorin Soprano biol. | 14 |
|  |  | Sorin Crown Bio | 99 |
|  |  | SJM Biocor | 17 |
|  |  | SJM Epic | 710 |
|  |  | SJM Trifecta | 1442 |
|  |  | SJM mechan. | 606 |
|  |  | Vascutek Aspire | 11 |
|  |  | other biol. valve | 338 |
|  |  | other mechan. valve | 175 |
|  |  | Rekonstruction | 4 |
|  |  |  |  |
