# Supplementary material for: Short- and Long-Term Outcomes After Transcatheter or Surgical Aortic Valve Replacement in Patients With Chronic Lung Disease: An Analysis From the German Aortic Valve Registry
Source: Interdiscip Cardiovasc Thorac Surg. 2025 Aug 18;40(8):ivaf189. doi: 10.1093/icvts/ivaf189 (PMC12396625; doi:10.1093/icvts/ivaf189)

Supplementary Figure 1: Patient selection process for patients with chronic lung disease from the GARY registry (final population A) and patient selection process for patients with chronic lung disease leading to propensity-score evaluated groups (final population B).

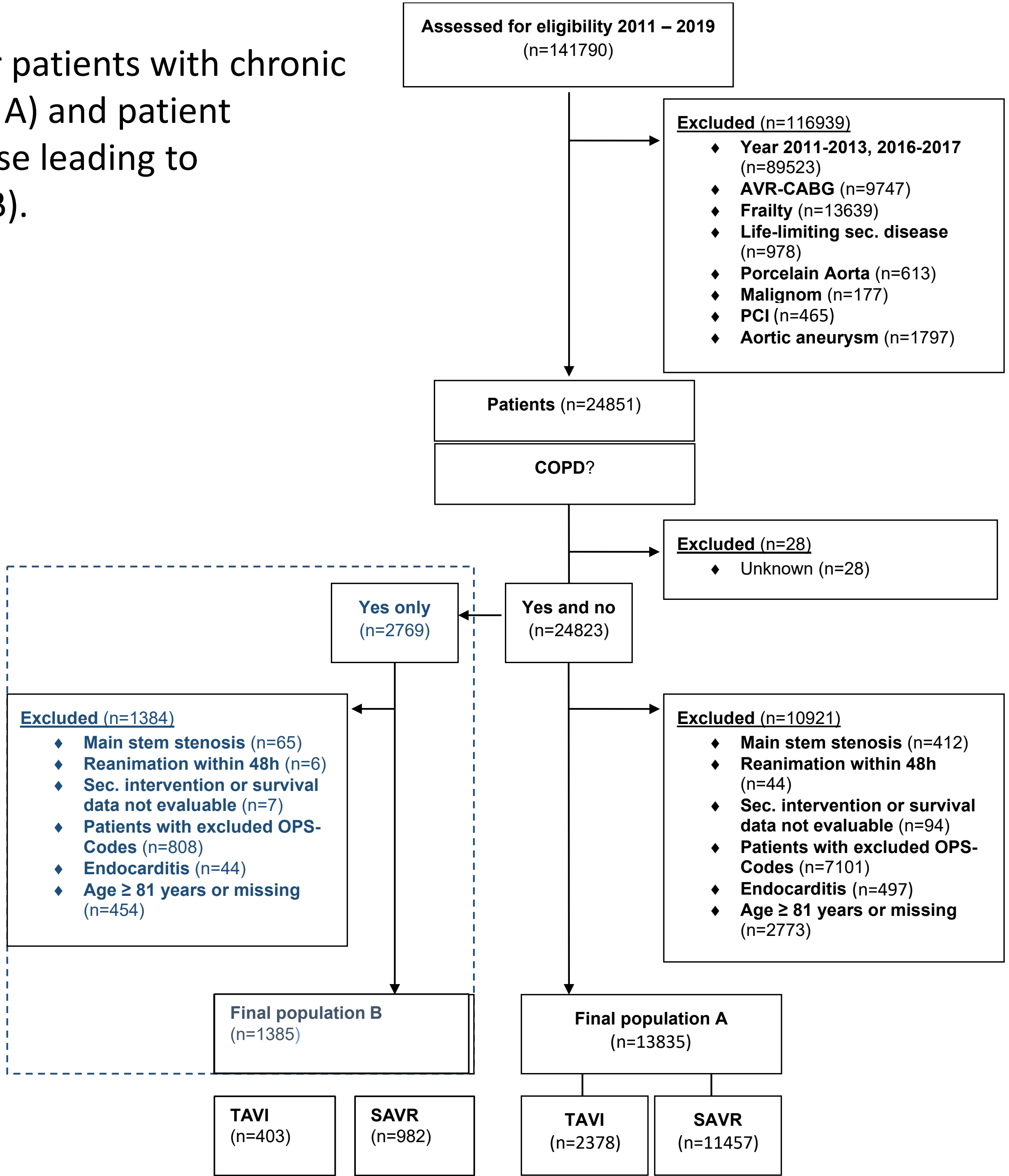

Abbreviations: COPD, chronic obstructive pulmonary disease; TAVI, transcatheter aortic valve implantation, SAVR; surgical aortic valve replacement; AVR-CABG, combined aortic valve replacement and coronary artery bypass grafting; PCI, percutaneous coronary intervention; OPS, German operations and procedures system ; Sec., secondary

Supplementary Figure 2: Absolute standardized mean differences using the unweighted evaluation (red) and the propensity score weighted data evaluation (black). he dotted horizontal lines marks standardized mean dfference of 0.1 and 0.2, respectively.

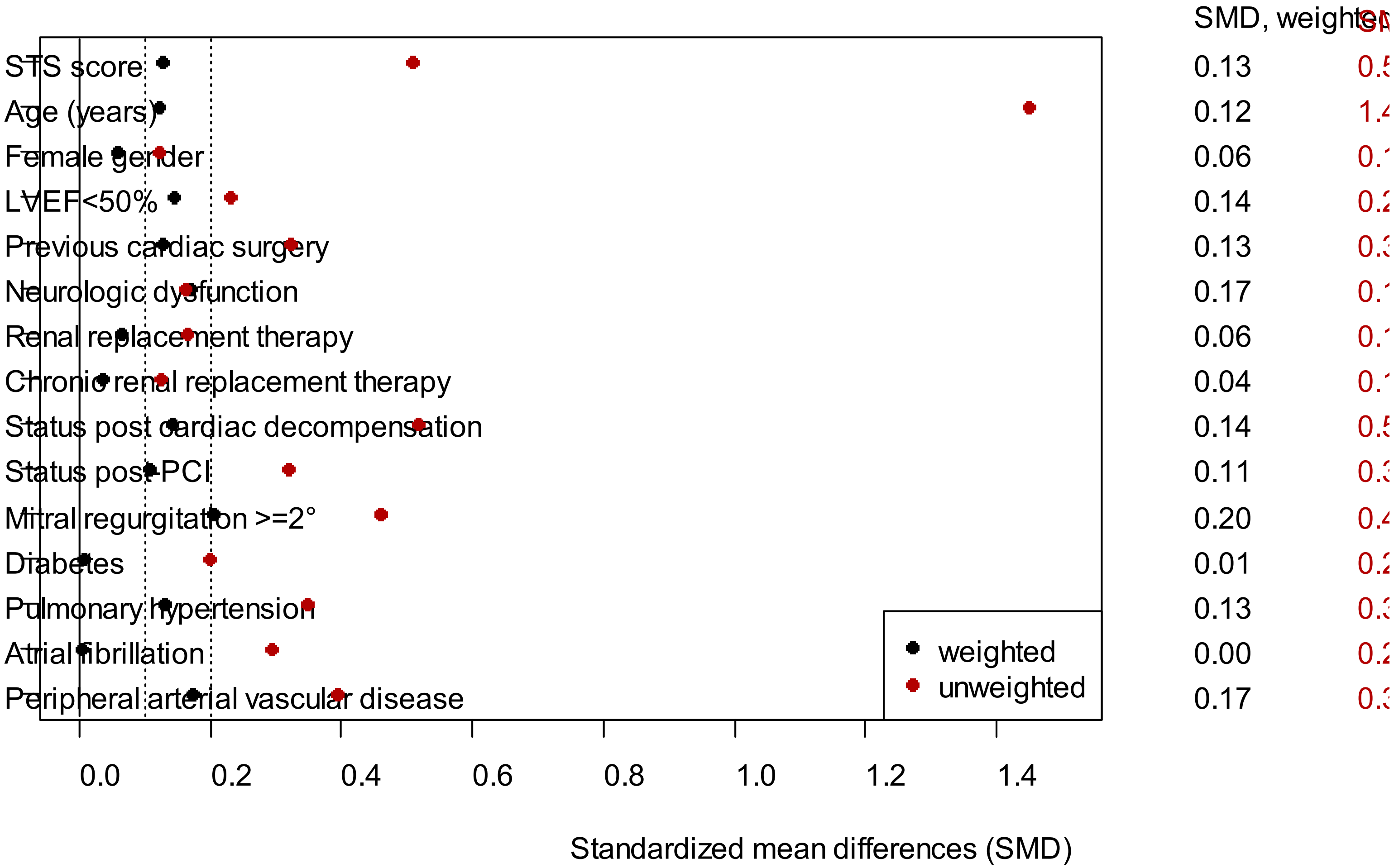

Abbreviations: LVEF, left ventricular ejection fraction; PCI, percutaneous coronary intervention; STS, Society of Thoracic Surgeons

Supplementary Figure 3: Absolute standardized mean differences using the unmatched and unweighted evaluation (red) and the propensity score matched data evaluation (black). The dotted horizontal lines marks standardized mean dfference of 0.1 and 0.2, respectively.

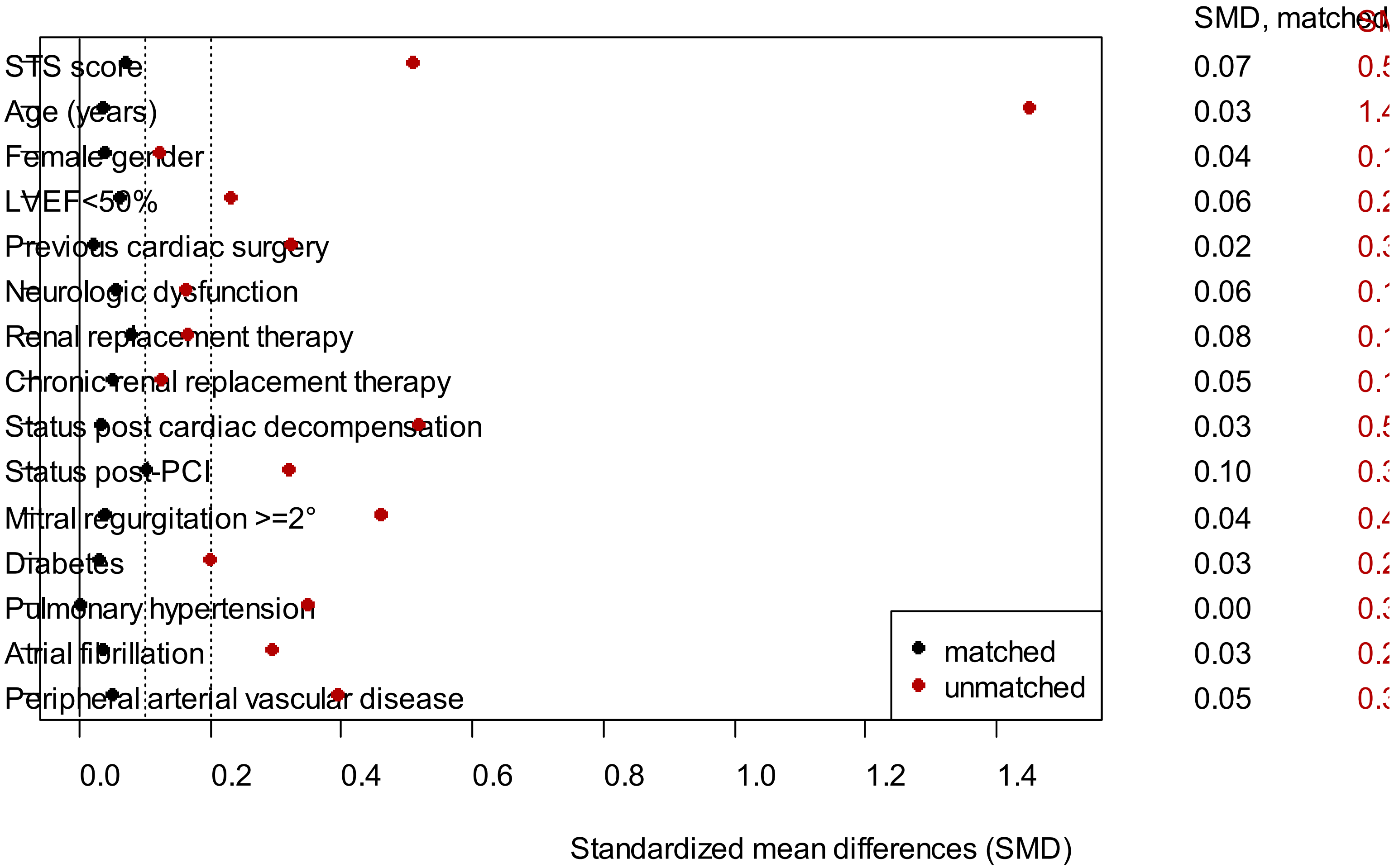

Abbreviations: LVEF, left ventricular ejection fraction; PCI, percutaneous coronary intervention; STS, Society of Thoracic Surgeons

Supplementary Figure 4: 5-year survival. Comparison of both adjusted methods: SAVR – weighted vs. TAVI (p=0.029) and SAVR, matched vs. TAVI, matched (p=0.003). As both analysis represent different populations, the observed differences are reasonable. The SAVR weighted analysis weighted the survival estimates so survival for SAVR treated patients in a population comparable to the TAVI population. The matched analysis stands – very roughly – for a intersection of the population of SAVR and TAVI patients, overall a population with a somewhat better prognosis.

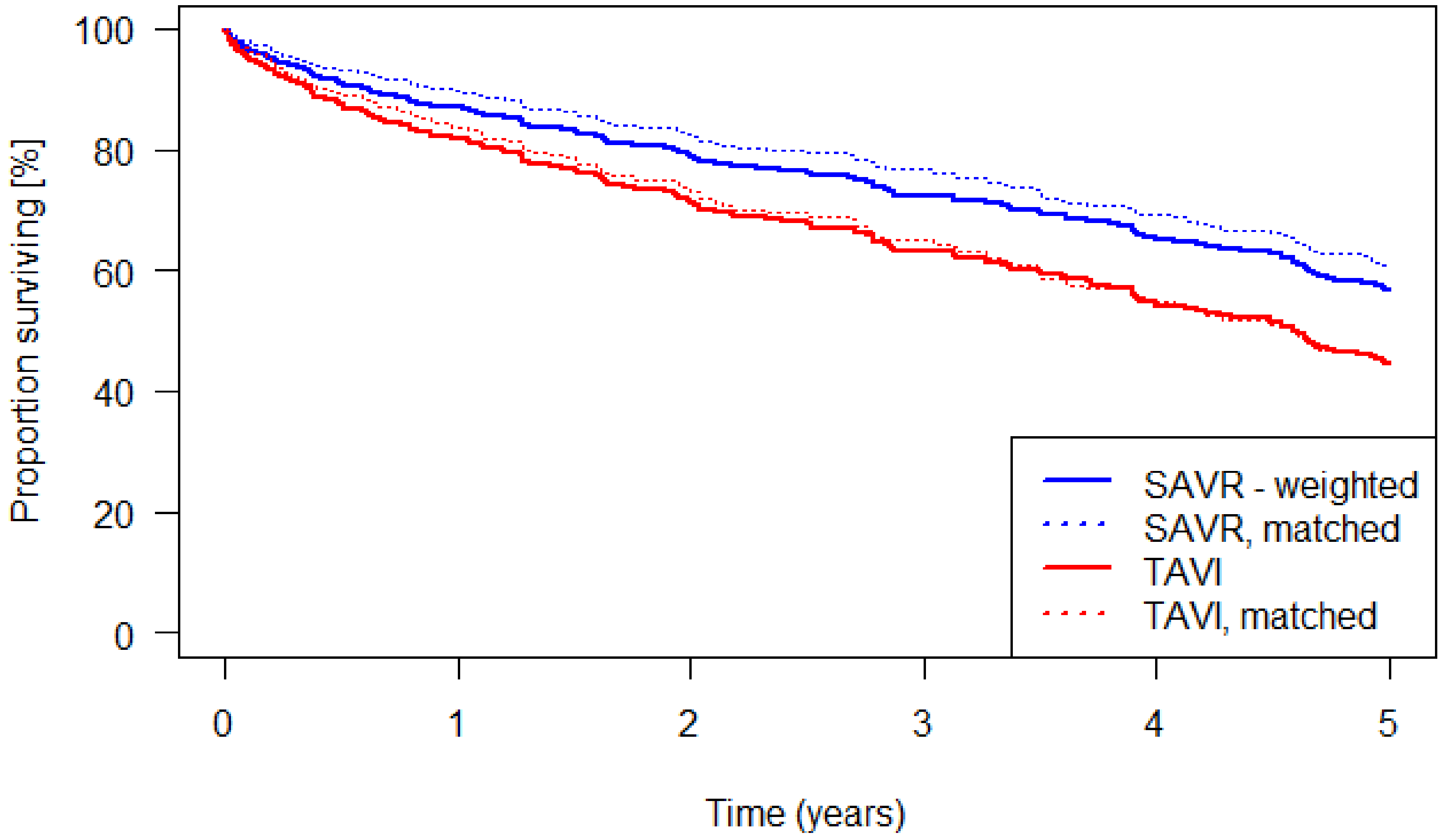

Supplement: ivaf189_Supplementary_Data [file ivaf189_supplementary_data.zip › Paper GARY lung study Supporting figures 13.06.25.pdf]
